# Physiological and Biochemical Screening of Chilli (*Capsicum annuum* L.) Genotypes to Short‐Term Waterlogging Stress at the Seedling Stage

**DOI:** 10.1155/sci5/4290084

**Published:** 2026-09-30

**Authors:** Md. Sabibul Haque, Zarin Shaima, Md. Fazle Rabbi, Hafsa Tasnim, Atia Iffat, Susmita Biswas, Md. Nesar Uddin, Md. Alamgir Hossain

**Affiliations:** ^1^ Department of Crop Botany Bangladesh Agricultural University Mymensingh 2202 Bangladesh bau.edu.bd

**Keywords:** *Capsicum*, chlorophyll fluorescence, leaf gas exchange, multivariate analysis, redox homeostasis, waterlogging tolerance

## Abstract

Waterlogging (WL) has emerged as a serious threat for chilli (*Capsicum annuum* L.) growth and productivity. A practical approach to overcome this challenge is to explore chilli germplasms with enhanced tolerance to WL stress. This study investigated the responses of 12 diverse chilli genotypes to short‐term WL stress and subsequent recovery at the seedling stage. The 35‐d‐old chilli seedlings were exposed to three growth conditions in pots: (i) control (no stress), (ii) WL (3‐d), and (iii) recovery (6‐d after WL), following a completely randomized design with three replications. WL stress significantly declined root‐shoot biomass, pigment contents, stomatal conductance (*g*_*s*_), transpiration rate (*E*), quantum yield of PSII (*Φ*_*P* *S* *I* *I*_), and electron transport rate (ETR) while markedly increased malondialdehyde (MDA), hydrogen peroxide (H_2_O_2_), proline contents, and total antioxidant capacity (TAC) in all tested genotypes. The genotypes varied significantly in response to the studied traits demonstrating tolerance and susceptibility. Depending on genotypes, total dry weight was reduced by 16%–36% under WL compared to control while only 8%–16% of the lost dry weight was recovered after a six‐day recovery. WL triggered severe impact on *g*_*s*_, reducing by 75%–95% across the genotypes compared to control. WL boosted leaf MDA by 30% in Sariakandi‐1 to 60% in BARI Morich‐3 compared to control, while after recovery, MDA levels decreased by 9%, 12%, and 15% in Premium, Current, and Sariakandi‐1, respectively. Under WL, the leaf proline and TAC in Current, Premium, and SK‐1 boosted by 72%, 80% and 82% and by 78%, 82%, and 83%, respectively, compared to control. Based on stress tolerance index (STI) of all measured traits, hierarchical clustering ranked Sariakandi‐1, Premium, BARI Morich‐5, and Current as relatively more tolerant to WL with comparatively better but partial recovery through the restoration of morpho‐physiological and biochemical attributes. Principal component analysis identified key traits as potential selection criteria for breeding WL‐tolerant chilli cultivars.

## 1. Introduction

Chilli (*Capsicum annuum* L.) plant is a member of the Solanaceae family and frequently used as an important quotidian spice in Bangladesh and worldwide [1]. The chilli fruits are abundant in capsaicin, carotenoids, flavonoids, vitamin C, vitamin E, malic acid, and other health beneficial substances [2]. Together with fresh consumption, it is also used as dry powder and for medicinal and ornamental purposes. Chilli is a foremost cash crop in Bangladesh and extensively cultivated in both winter (Rabi) and summer (Kharif) seasons. During the Kharif and Rabi seasons in 2023‐2024, chilli was cultivated in areas of 53,161 and 191,521 acres of land with the production of 186,404 and 56,197 MT, respectively, in Bangladesh [3]. Chilli production particularly in Kharif season is often curtailed by waterlogged conditions due to heavy monsoon rainfall, flash flood, poor soil drainage, rush of water, flat topography, and so on [4]. In the year 2020‐2021, the total loss in Kharif chilli production was 378 MT in Bangladesh due to waterlogging (WL) caused by excessive rainfall and flooding [3]. Chilli plants exhibit sensitivity to WL conditions due to their shallow root architecture resulting in substantial production loss [5–7]. WL may cause death of chilli plants within 48 h if proper drainage is not timely applied [8].

WL eradicates air from the soil pores, restricting oxygen availability (hypoxia or anoxia) in the rhizosphere and thus suppresses roots’ aerobic respiration, decreases root activity, cuts ATP production, enhances the accumulation of toxic by‐products, and disrupts the plant’s entire metabolic processes [9–12]. The key gas exchange attributes such as stomatal conductance, transpiration rate, and photosynthesis rate are greatly impeded due to WL stress in plants including chilli [13, 14]. Long‐term WL impaired chlorophyll synthesis and declined light absorption leading to a significant reduction in the rate of photosynthesis [15]. Moreover, the Rubisco activity is further declined affecting photosynthesis in plants [16]. WL causes curling, wrinkling, senescence, and yellowing of pepper leaves inhibiting gas exchange traits and thereby decreases leaf area, plant height, and root biomass [14, 17]. During WL, insufficient O_2_ leads to an increase in intracellular reactive oxygen species (ROS) via the disruption of the electron transport chain (ETC) in mitochondria [18] leading to lipid peroxidation and cell membrane damage [19]. ROS generation further exacerbates through hormonal imbalances like enhanced production of ethylene and abscisic acid and mediates stomatal closure and adventitious root formation [20, 21].

Almost all plants suffer intensively under waterlogged conditions; however, they adapt through series of morphological, physiological, and molecular modifications under such environmental stress [22–25]. WL alters the morphology of plants such as adventitious root formation, prompt elongation of apical meristems, and air film creation in the upper cuticle [26]. The most physiological response to WL in plants is the development of aerenchyma which provides O_2_ as well as discharges CO_2_ and toxic volatile substances from waterlogged tissues [27, 28]. Plants can rely on antioxidants to maintain the dynamic balance of ROS, thus reducing the extent of oxidative damage [29]. The enzymatic antioxidants like catalase (CAT), ascorbate peroxidase (APX), guaiacol peroxidase (GPX), and superoxide dismutase (SOD) have been reported to increase under WL treatments in plants [30]. Plants balance the synthesis and transport of phytohormones and cope with WL stress by embracing an orchestrated set of physiological adjustments, which are regulated by an elaborate hormonal signaling network [31]. The activities of SOD, GPX, CAT, peroxidase (POD), and alcohol dehydrogenase (ADH) in WL‐tolerant pepper markedly boosted after 5 days of water treatment [32].

Plant tolerance to WL depends on species or cultivar susceptibility, developmental stage at the time of WL, and the duration of WL [33]. The flooding tolerance of several *Capsicum* species diverges because of their unique physiological and genetic features [34–36]. *Capsicum frutescens* showed improved tolerance than *C. chinense* in response to stomatal conductance and photosynthetic rate [37]. Post‐WL or recovery period is also crucial, and the retrieval rate of morphological and physiological WL‐mediated disorders are largely species‐ or cultivar‐specific [36, 38, 39]. In Bangladesh, WL at both early and later developmental stages particularly in Kharif season severely interrupts chilli production, leading to limited fruit supply and consequently sharp rises in market prices. These erratic WL or flooding events also impose substantial economic suffering on farmers. Therefore, development of WL‐resilient chilli cultivars is decisive for sustaining chilli production, particularly in regions characterized by poor soil drainage, persistent heavy rainfall, and intense flooding. Bangladesh possesses a wide diversity of chilli germplasms, including local landraces, inbred lines, and commercial hybrid cultivars that are cultivated across different agroecological regions. Flash flood‐ or heavy rainfall‐induced WL in chilli‐growing regions is generally transient, with inundation often lasting only a few days before water recedes. Thus, assessment of the existing chilli genotypes to short‐term waterlogged conditions followed by a quick recovery is necessary. However, the extent of WL tolerance and post‐stress recovery of commercially available and extensively cultivated local, inbred, and hybrid chilli cultivars has not yet been comprehensively and systematically examined under Bangladeshi conditions. In particular, comparative assessments integrating morphological, physiological, and biochemical responses to WL stress, as well as post‐WL recovery of these cultivars at the seedling stage, remain largely unexplored. Therefore, the present study was conducted to evaluate short‐term response of diverse chilli genotypes (local, inbred, and hybrid) to WL and subsequent recovery capacity by integrating morphological, time‐course physiological, and biochemical attributes. The findings are expected to identify potential tolerant chilli genotypes and provide a scientific basis for future breeding programs and the development of WL‐resilient chilli cultivars.

## 2. Materials and Methods

### 2.1. Plant Materials and WL Treatments

The experiment was carried out in the greenhouse of the Agrometeorology Department and the Plant Physiology Laboratory of the Crop Botany Department, Bangladesh Agricultural University, Mymensingh, Bangladesh. A total of 12 chilli genotypes (four hybrid, four inbred, and four local) were collected and used in this study (Table 1).

**TABLE 1:** Names, types, and sources of 12 chilli genotypes used in this study.

SL	Genotype	Symbol used	Type	Sources
01	Heera	—	Hybrid	Supreme Seed Company Ltd., Bangladesh
02	Premium	—	Hybrid	Lal Teer Seed Ltd., Bangladesh
03	Bizli Plus 2020	Bizli	Hybrid	AR Malik Seeds Pvt Ltd., Bangladesh
04	Namdhari	—	Hybrid	Namdhari Seed Pvt Limited, India
05	BARI Morich‐1	BM‐1	Inbred	SRSc, BARI, Lalmonirhat, Bangladesh
06	BARI Morich‐3	BM‐3	Inbred	SRSc, BARI, Lalmonirhat, Bangladesh
07	BARI Morich‐4	BM‐4	Inbred	SRSc, BARI, Lalmonirhat, Bangladesh
08	BARI Morich‐5	BM‐5	Inbred	SRSc, BARI, Lalmonirhat, Bangladesh
09	Sariakandi‐1	SK‐1	Local	Bogura, Bangladesh
10	Sariakandi‐2	SK‐2	Local	Bogura, Bangladesh
11	Current	—	Local	Lalmonirhat, Bangladesh
12	Chilmari	—	Local	Chilmari, Bangladesh

Abbreviation: SRSc = Spices Research Sub‐Center; BARI = Bangladesh Agricultural Research Institute.

The collected seeds of all genotypes were primed with distilled water for 12 h and then dried overnight at room temperature to retain primary moisture levels. Later, seeds were surface‐sterilized with Vitavax‐200 @1.5 g kg^−1^. The seeds were then sown in plastic pots (20 cm in diameter) with sterilized sand for germination with proper watering. The 14 days old seedlings were then transferred to another plastic pot (20 cm × 18 cm − diameter × height) filled with 3 kg of field soil per pot. All the plants were properly irrigated and performed other intercultural operations before being exposed to WL stress. The soils are mixed with cow dung in a ratio of 1:3. The chemical fertilizers such as urea, triple super phosphate (TSP), and muriate of potash (MoP) were applied to the soils in each pot at the rates of 0.25, 0.15, and 0.15 g, respectively. Each pot contained five seedlings and was considered a single experimental unit, resulting in 15 seedlings per treatment across three pots (replicates). The five seedlings within each pot were maintained to provide representative biological material for morpho‐physiological and biochemical measurements, while the pot was treated as the experimental unit for statistical analysis. After 35 days of sowing (DAS), the pots of each genotype were divided into three groups such as (i) control (no stress) (ii) WL stress, and (iii) recovery (Rec). The WL stress was applied for 3 days by keeping a water height of 3–5 cm above the soil surface. The recovery pots had also remained under WL conditions for 3 days and then both WL and recovery pots were observed for a recovery period of 6 days by subsequent withdrawal of WL through draining out of water from the pots. The duration of the WL period was selected to simulate the transient nature of WL events commonly associated with flash floods and heavy rainfall, where standing water in chilli fields often persists for only a short period before receding. Moreover, Rob [8] reported that chilli seedlings are highly susceptible to WL and may not survive beyond 48 h of continuous flooding. Considering this high sensitivity, extending the WL period beyond 3 days was expected to substantially increase seedling mortality, thereby compromising subsequent morphological, physiological, and biochemical assessments. As limited or no previous information regarding recovery period was available, except Molla et al. [5], the recovery period of 6 days was purposely chosen to provide a preliminary assessment of the immediate resilience and early recovery responses of chilli seedlings following transient WL. Therefore, a 3‐day WL treatment followed by a 6‐day recovery period was considered appropriate to imitate a realistic field scenario while ensuring reliable experimental observations. The experiment was laid out in a two‐factorial (genotype and WL stress) completely randomized design (CRD) with three replicates (pots). Therefore, a total of 108 pots were used (genotype × stress × replication; 12 × 3 × 3). All the pots were kept in a greenhouse chamber with controlled environmental conditions. The plants were illuminated by approximately 800 μmol m^−2^ s^−1^ photosynthetic photon flux density (PPFD) from a combination of solar radiation and artificial solar lamps mounted inside the chamber. The temperature was set by 35/25°C (day/night) with a relative humidity (RH) of 60%–70%. The entire experimental setup is illustrated in Figure 1.

**FIGURE 1:**
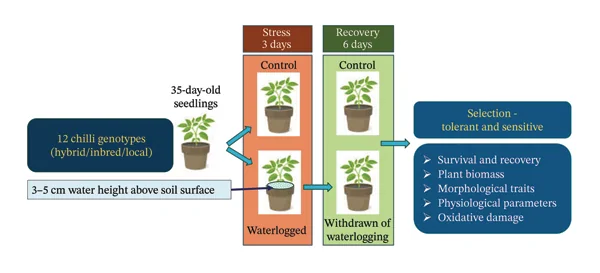
35‐day‐old seedlings of 12 diverse chilli genotypes were exposed to control and waterlogged (WL) conditions. After 3 days of waterlogging, the seedlings were kept for a recovery period of 6 days by draining out excess water from the pots. The morpho‐physiological and biochemical data of chilli seedlings were recorded at different days of WL and recovery phases for the selection of tolerant and sensitive genotypes.

### 2.2. Data Collection

#### 2.2.1. Morphological Attributes

The data recording for morphological traits was performed in two phases: after three days of WL stress (38 DAS) and six days (44 DAS) of recovery period. The roots and shoots of three seedlings of each treatment (one from each replicated pot) were carefully separated after the seedlings were meticulously extracted from the plant. The average values were employed for data analysis, and six seedlings were randomly selected (two from each container) for morphological measurements. Using a 1 m ruler, the root length (RL) and shoot length (SL) were measured in centimeters. A digital weighing balance was used to measure the fresh weight of the root and shoot (RFW and SFW in mg). The samples were oven‐dried for 72 h at 70°C to determine the dry weight of the roots (RDW) and shoots (SDW). Using the aggregate of RFW and SFW and RDW and SDW, respectively, the seedlings’ total fresh weight (TFW) and total dry weight (TDW) were computed in milligrams. RDW over SDW was the measure used to determine the root‐shoot ratio (RSR).

#### 2.2.2. Leaf Gas Exchange and Chlorophyll Fluorescence Measurements

The stomatal conductance (*g*_*s*_, mol m^−2^ s^−1^), rate of transpiration (*E*, mmol m^−2^ s^−1^), quantum yield of photosystem (PS) II (*Φ*_*P* *S* *I* *I*_), and electron transport rate (ETR; μmol m^−2^ s^−1^) were recorded on day 0, 1, 2, and 3 (S0–S3) of WL stress and on third and sixth day (R3 and R6) of recovery period. Three leaves of three plants from each treatment (of all genotypes) were tagged and the gas exchange and chlorophyll fluorescence measurements were performed on the same leaves at different days of stress and recovery by a portable porometer cum fluorometer (LI‐600, LI‐COR Environmental, Lincoln, USA).

#### 2.2.3. Leaf Pigments Determination

Fresh leaf samples were collected from the seedlings under stress and recovery conditions for pigment analysis. The chlorophyll a (Chl a), chlorophyll b (Chl b), total chlorophyll (total Chl), and total carotenoids (total Car) contents were determined by the protocol described by Lichtenthaler [40]. Around 50 mg of freshly collected leaf sample was dipped in 10 mL of 80% acetone and left for 7 days for adequate extraction of pigments in dark conditions. All data were calculated from the absorbance readings at 470, 646.6, and 663.2 nm wavelengths by a UV–Vis spectrophotometer (T6U, Persee, PG Scientific INC., CA, USA) for the calculation of pigment contents (mg g^−1^ FW).

#### 2.2.4. Determination of Malondialdehyde (MDA) Contents

The thiobarbituric acid reactive substances (TBARS) assay was performed to estimate the MDA content as a measure of lipid peroxidation in leaf samples [41]. Approximately 0.1 g of fresh leaf samples were pulverized with a mortar and pestle using liquid nitrogen and then homogenized with 0.1% trichloroacetic acid (TCA) in a 2 mL centrifuge tube. The homogenized components were centrifuged at 12,000 rpm for 15 min. The supernatants were transferred into a fresh tube, and a reaction mixture of 1.5 mL was prepared by mixing 0.5% thiobarbituric acid (TBA) that contained 20% TCA. After 15 min at 90°C in a water bath, the tubes were immediately immersed in ice for 10 min to halt the reaction. A UV–Vis spectrophotometer (T6U, Persee, PG Scientific INC., CA, USA) was used to evaluate the optical density of the supernatants at 532 and 600 nm against a blank that included all reagents except the biological sample. The attenuation coefficient of 155 mM^−1^ cm^−1^ was used to calculate the MDA concentrations (nmol g^−1^ FW) after subtracting the absorbance values at 600 nm from the 532 nm.

#### 2.2.5. Determination of Leaf H_2_O_2_ Contents

The H_2_O_2_ contents in the collected leaf samples were estimated using the protocol described by Alexieva [42]. About 100 mg fresh leaf samples were ground in liquid nitrogen with the use of a pre‐cooled mortar and pestle. The samples were then homogenized with 0.1% TCA and centrifuged for 15 min at 12,000 rpm at 4°C. A reaction mixture of 1.5 mL was prepared with the plant extract, 1 M KI, and a 10 mM KH_2_PO_4_ buffer. The mixture was then kept in darkness for an hour to facilitate the reaction. The absorbances from the samples were taken at 390 nm using a spectrophotometer. The H_2_O_2_ content (μmol g^−1^ FW) was calculated using a standard curve prepared from known concentrations of H_2_O_2_.

#### 2.2.6. Measurement of Proline Content and Total Antioxidant Capacity (TAC)

The leaf proline content was measured using the protocol described by Carillo [43] with minor modifications [44]. Approximately 50 mg of fresh leaf samples were ground with liquid nitrogen, and the leaf powder was mixed with 70% ethanol. After centrifuging the homogenates at 12,000 rpm for 5 min, the reaction mixture was prepared with 1% ninhydrin (*w/v*) in 60% acetic acid (*v/v*) and 20% ethanol (*v/v*) including the plant extract. The sample tubes were then placed in a water bath at 95°C for 30 min to allow the reaction. The samples were then cooled in room temperature and absorbances were taken at 520 nm using a spectrophotometer. The proline content (μg g^−1^ FW) was estimated using a standard curve of standard proline of several known concentrations. The TAC was analyzed following the protocol described by Prieto [45] with slight modifications [46]. The TAC is expressed as mg equivalent AA g^−1^ FW, where AA elaborates ascorbic acid.

### 2.3. Statistical Data Analysis

The statistical analysis of collected data was performed using open‐source statistical software R v. 4.0.5 [47], accessed in April 2024. The normal distribution of the data was assessed using the Shapiro–Wilk test, while the homogeneity of variances among treatment groups was examined using Levene’s test. The two‐way (genotypes and WL stress) analysis of variance (ANOVA) was done to compare among the genotypes and the treatments. The multiple comparisons of the means of treatment combinations were accomplished by the Tukey HSD test with a significant level of *p* < 0.05. The stress tolerance index (STI) values for each trait were calculated as stress values/control values × 100. The normalized STI values of WL and recovery data were utilized to create the two‐way hierarchical clustering heatmaps using *ComplexHeatmap* package in R program. The principal component analysis (PCA) biplot was constructed using *fviz_pca* function under *factoextra* package of R.

## 3. Result

### 3.1. Morphological Attributes

WL stress significantly affected the morphological attributes in all tested chilli genotypes (Supporting Table 1, Tables 2, and 3). However, the genotypes displayed variable responses in terms of WL stress conferring to their adaptability under WL and recovery periods. In WL stress, the longest root length (LRL) among the genotypes ranged from 6.1 cm (Namdhari) to 9.5 cm (Bizli) and percent reduction of LRL in respect to control ranged from 5% (Premium) to 45% (Namdhari) (Table 2). While after 6 days of recovery phase, the highest percent increase in LRL was noticed in Namdhari (16%) followed by BM‐1 (14%) compared to WL. In case of SL under WL, percent reduction varied from 8% (Current) to 25% (Heera) compared to control, and after 6 days of recovery, percent increase in SL was highest in Heera (16%) compared to WL (Table 2). Root fresh weight (RFW) was reduced by 52%–21%, respectively, in Namdhari and Current in WL compared to the control, and after recovery, it increased by 7% (Bizli) to 21% (SK‐1). Besides, the genotypes Namdhari, BM‐5, and Heera demonstrated better recovery in relation to RFW scoring 16%, 15%, and 14% enhancement, respectively. Similarly, shoot fresh weight (SFW) was reduced by 19% (Premium) to 38% (SK‐2) and, subsequently, the SFW recovered by 6% (Namdhari) to 21% (SK‐2) (Table 2). The percentage reduction in root (RDW) and shoot dry weight (SDW) ranged between 23% (Premium) and 63% (Bizli) and between 14% (Premium) and 37% (BM‐1), respectively (Table 3). The recovery rate of RDW was higher in SK‐2 (25%) followed by BM‐1 (17%) in comparison to WL. The WL‐mediated maximum decline in TFW and TDW was recorded in SK‐2 (38%) and BM‐1 (36%) while these genotypes tended to manage a faster recovery in relation to plant biomass (Table 3). Premium and Current genotypes exhibited lower reductions in terms of fresh and dry weight implying tolerance to WL stress (Table 3).

**TABLE 2:** Longest root length (LRL), shoot length (SL), root fresh weight (RFW), and shoot fresh weight (SFW) of 12 chilli genotypes grown in control, waterlogging (WL), and post‐stress recovery (Rec) conditions.

Genotypes	Treatments	LRL (cm plant^−1^)	SL (cm plant^−1^)	RFW (mg plant^−1^)	SFW (mg plant^−1^)
SK‐1	Control	6.6 ± 0.4 klm	14.9 ± 1.1 a	147.5 ± 0.5 h‐k	1361 ± 64 bcd
	WL	6.0 ± 0.5 m	13.5 ± 1.5 ab	94.5 ± 4.5 nop	1031 ± 97 g‐j
	Rec	6.3 ± 0.3 sl	15.0 ± 1 a	119 ± 11 k‐o	1185 ± 25 efg

SK‐2	Control	8.2 ± 0.1 c‐l	11.2 ± 0.8 cde	152 ± 10 hij	988.5 ± 27.5 hij
	WL	7.1 ± 0.5 j‐m	9.9 ± 0.9 d‐i	86.5 ± 5.5 p	616 ± 12 o‐r
	Rec	7.2 ± 0.4 j‐m	11.1 ± 0.9 cde	98.5 ± 11.5 m‐p	776.5 ± 26.5 lmn

Namdhari	Control	11.0 ± 0.4 ab	11.1 ± 0.1 cd	273 ± 13 bc	1596 ± 9 a
	WL	6.1 ± 1.0 m	10.4 ± 1.3 c‐h	130 ± 10 i‐m	1298.5 ± 185.5 b‐e
	Rec	7.2 ± 0.3 j‐m	10.0 ± 0.5 d‐l	155 ± 10 ghi	1386.5 ± 15.5 b

Current	Control	9.7 ± 0.2 b‐f	11.0 ± 0.5 c‐f	196.5 ± 8.5 ef	1109 ± 41 fgh
	WL	8.1 ± 1.6 c‐l	10.1 ± 1.1 d‐h	153.5 ± 10.5 hij	827 ± 39 klm
	Rec	8.6 ± 0.2 c‐k	10.7 ± 0.2 c‐g	167.5 ± 7.5 fgh	917 ± 23 jkl

Heera	Control	10.0 ± 1.5 a‐d	14.0 ± 0.6 ab	299.5 ± 12.5 ab	1734 ± 44 a
	WL	7.1 ± 0.9 j‐m	10.6 ± 0.3 c‐g	201 ± 19 e	1222.5 ± 20.5 c‐f
	Rec	7.8 ± 0.3 f‐m	12.5 ± 1.3 bc	233.5 ± 24.5 d	1374.5 ± 94.5 bc

Bizli	Control	11.7 ± 0.5 a	10.4 ± 0.6 c‐h	286.5 ± 19.5 ab	1423 ± 14 b
	WL	9.5 ± 1.6 b‐g	9.2 ± 0.1 d‐j	174 ± 4 e‐h	979 ± 28 h‐k
	Rec	9.3 ± 0.3 b‐i	9.0 ± 0.5 e‐j	186.5 ± 6.5 efg	1127 ± 80 fgh

Chilmari	Control	10.1 ± 0.6 abc	10.6 ± 0.7 c‐g	125 ± 6 i‐n	947.5 ± 4.5 ijk
	WL	6.6 ± 0.9 klm	9.2 ± 1.1 d‐j	95.5 ± 2.5 nop	604.5 ± 14.5 o‐r
	Rec	7.3 ± 0.3 j‐m	9.1 ± 0.1 d‐j	105 ± 5 l‐p	690 ± 40 m‐p

Premium	Control	7.5 ± 0.2 g‐m	9.9 ± 0.7 d‐i	317 ± 7 a	`1219 ± 38 def
	WL	7.1 ± 0.5 j‐m	8.8 ± 0.7 f‐k	249 ± 9 cd	992 ± 21 hij
	Rec	7.4 ± 0.1 h‐m	8.9 ± 0.7 f‐k	279 ± 10 bc	1088.5 ± 18.5 f‐i

BM‐5	Control	9.4 ± 0.4 b‐h	7.1 ± 0.2 j‐m	132.5 ± 7.5 i‐l	602 ± 5 o‐r
	WL	7.7 ± 0.7 f‐m	6.2 ± 0.5 m	85.5 ± 3.5 p	466.5 ± 7.5 rs
	Rec	8.0 ± 0.5 d‐m	6.5 ± 0.2 lm	100 ± 10 m‐p	535 ± 7 qrs

BM‐4	Control	8.6 ± 0.3 c‐k	8.7 ± 0.5 g‐l	122.5 ± 12.5 j‐n	659 ± 25 n‐q
	WL	7.6 ± 0.4 g‐m	7.1 ± 0.6 j‐m	77.5 ± 1.5 p	446.5 ± 18.5 s
	Rec	7.9 ± 0.2 e‐m	7.5 ± 0.2 j‐m	87.5 ± 7.5 op	537 ± 30 p‐s

BM‐3	Control	9.9 ± 0.4 a‐e	7.9 ± 0.4 i‐m	132.5 ± 7.5 i‐l	663.5 ± 16.5 n‐q
	WL	7.8 ± 0.5 f‐m	6.4 ± 0.1 m	82.5 ± 3.5 p	483 ± 15 rs
	Rec	8.0 ± 0.3 e‐m	6.7 ± 0.2 klm	100 ± 10 m‐p	526.5 ± 24.5 qrs

BM‐1	Control	9.1 ± 0.6 b‐j	8.2 ± 0.2 h‐m	130 ± 4 i‐m	720 ± 10 mno
	WL	6.3 ± 0.1 lm	7.4 ± 0.2 j‐m	83 ± 6 p	564 ± 16 p‐s
	Rec	7.3 ± 0.3 i‐m	7.6 ± 0.1 j‐m	97 ± 5 nop	610 ± 10 o‐r

*Note:* Values are mean ± SD. In a column, treatment means with dissimilar letters are significant at 5% levels of probability.

**TABLE 3:** Shoot dry weight (SDW), root dry weight (RDW), total fresh weight (TFW), and total dry weight (TDW) of 12 chilli genotypes grown in control, waterlogging (WL), and post‐stress recovery (Rec) conditions.

Genotypes	Treatments	SDW (mg plant^−1^)	RDW (mg plant^−1^)	TFW (mg plant^−1^)	TDW (mg plant^−1^)
SK‐1	Control	168.1 ± 7.7 hij	27.7 ± 3.6 g‐j	1509 ± 65 def	195.8 ± 4.1 fgh
	WL	139.6 ± 6.6 kl	17.0 ± 2.9 l‐o	1126 ± 93 jkl	156.6 ± 9.5 jk
	Rec	152.5 ± 7.5 ijk	19.6 ± 3.1 j‐o	1304 ± 14 ghi	172.1 ± 10.6 hij

SK‐2	Control	133.6 ± 9.8 kl	26.7 ± 2.9 g‐k	1141 ± 38 jkl	160.25 ± 12.65 jk
	WL	97.9 ± 8.2 nop	13.9 ± 1.4 no	703 ± 18 o‐r	111.8 ± 6.8 l‐p
	Rec	102.5 ± 7.5 m‐p	18.6 ± 6.9 j‐o	875 ± 15 mn	121.1 ± 14.4 l‐o

Namdhari	Control	238.0 ± 13.7 bc	67.7 ± 5.0 a	1869 ± 4 b	305.7 ± 18.7 b
	WL	187.4 ± 26.9 fgh	31.8 ± 2.0 e‐i	1429 ± 176 efg	219.23 ± 28.93 efg
	Rec	202.5 ± 7.5 def	39.7 ± 3.0 cde	1542 ± 6 de	242.15 ± 10.45 e

Current	Control	158.1 ± 6.5 ijk	33.5 ± 1.9 e‐h	1306 ± 50 ghi	191.5 ± 8.3 ghi
	WL	135.0 ± 2.0 kl	24.1 ± 0.6 h‐m	981 ± 50 lm	159.1 ± 2.6 jk
	Rec	147.5 ± 2.5 jkl	27.4 ± 0.9 g‐j	1085 ± 31 jkl	174.85 ± 3.35 hij

Heera	Control	289.5 ± 3.5 a	52.1 ± 2.3 b	2034 ± 57 a	341.55 ± 1.25 a
	WL	241.3 ± 12.7 bc	34.4 ± 2.7 d‐g	1424 ± 2 efg	275.7 ± 10 cd
	Rec	261.5 ± 6.5 b	38.4 ± 1.6 c‐f	1608 ± 119 cd	299.9 ± 8.1 bc

Bizli	Control	226.1 ± 5.5 cd	66.5 ± 6.4 a	1710 ± 6 bc	292.55 ± 11.85 bc
	WL	174.2 ± 10.1 ghi	25.0 ± 1.4 h‐l	1153 ± 24 ijk	199.15 ± 8.75 fgh
	Rec	190.0 ± 8.0 fgh	33.4 ± 2.9 e‐h	1314 ± 87 ghi	223.35 ± 5.15 ef

Chilmari	Control	126.2 ± 7.7 lm	32.0 ± 2.0 e‐i	1073 ± 2 kl	158.1 ± 5.7 jk
	WL	90.8 ± 3.2 n‐q	14.9 ± 0.5 mno	700 ± 17 o‐r	105.65 ± 3.65 m‐p
	Rec	103.0 ± 5.0 m‐p	19.1 ± 1.2 j‐o	795 ± 35 nop	122.1 ± 3.8 l‐o

Premium	Control	222.9 ± 2.8 cde	61.7 ± 6.5 a	1536 ± 45 de	284.6 ± 9.3 bc
	WL	191.0 ± 10.5 fgh	43.8 ± 2.2 bcd	1241 ± 30 hij	234.75 ± 12.65 e
	Rec	200.0 ± 5.0 efg	47.5 ± 2.5 bc	1368 ± 29 fgh	247.5 ± 2.5 de

BM‐5	Control	93.3 ± 6.1 n‐q	39.4 ± 0.8 cde	735 ± 13 n‐q	132.65 ± 6.85 klm
	WL	78.4 ± 1.6 opq	24.7 ± 1.3 h‐l	552 ± 11 rs	103.1 ± 2.9 m‐p
	Rec	83.5 ± 1.5 n‐q	29.3 ± 3.6 f‐i	635 ± 17 p‐s	112.75 ± 2.05 l‐o

BM‐4	Control	90.4 ± 2.9 n‐q	23.0 ± 0.5 i‐n	782 ± 38 n‐q	113.4 ± 3.4 lmno
	WL	71.1 ± 1.1 q	11.9 ± 0.1 o	524 ± 20 s	82.9 ± 1.1 p
	Rec	78.0 ± 3.0 pq	15.0 ± 1.5 mno	625 ± 23 qrs	92.95 ± 4.45 op

BM‐3	Control	106.5 ± 4.5 mn	33.1 ± 2.6 e‐h	796 ± 9 nop	139.55 ± 7.05 kl
	WL	78.9 ± 3.6 opq	22.9 ± 1.6 g‐l	566 ± 19 rs	101.8 ± 2 nop
	Rec	88.0 ± 3.0 n‐q	25.7 ± 2.2 i‐n	627 ± 35 qrs	113.7 ± 0.8 l‐o

BM‐1	Control	136.4 ± 6.4 kl	25.6 ± 2.5 g‐l	850 ± 6 mno	161.95 ± 3.95 ijk
	WL	86.0 ± 4.7 n‐q	17.5 ± 2.2 k‐o	647 ± 22 p‐s	103.45 ± 6.85 m‐p
	Rec	104.0 ± 6.0 mno	19.1 ± 1.2 j‐o	707 ± 15 o‐r	123.05 ± 7.15 lmn

*Note:* Values are mean ± SD. In a column, treatment means with dissimilar letters are significant at 5% levels of probability.

### 3.2. Physiological Attributes

#### 3.2.1. Stomatal Conductance (**g**_**s**_) and Transpiration Rate (*E*)

The WL stress significantly hindered the gaseous exchange of all chilli genotypes, which were partially improved during the recovery phase (Supporting Table 1, Figures 2 and 3). Stomatal conductance (*g*_*s*_) declined abruptly among the genotypes with the prolongation of stress period (Figure 2). The maximum percent reduction in *g*_*s*_ over control was observed in Bizli (97%) followed by Namdhari, Heera, Chilmari, and BM‐3 (96%), along with BM‐4 and SK‐2 (95%). On the contrary, 6‐day recovery period showed a positive increasing trend of *g*_*s*_ in all genotypes compared to WL and control but none of the genotypes exhibited a complete recovery. The faster recovery rate was noticed in Heera (40%) followed by Premium (29%), Current (27%) and Namdhari (26%) while poor recovery in *g*_*s*_ was apparent in Bizli (2%) and BM‐1 (4%) (Figure 2).

**FIGURE 2:**
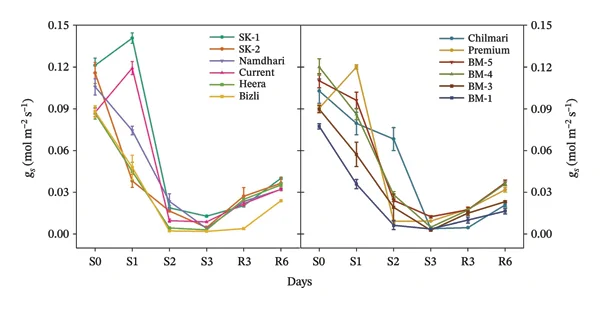
Changes in stomatal conductance (*g*_*s*_; mol m^−2^ s^−1^) in the seedlings of 12 chilli genotypes on different days of waterlogging and recovery period. S0 denotes the day before WL stress; S1, S2, and S3 are the three consecutive days of WL stress; R3 and R6 denote third and sixth days of recovery period. Vertical bars are SD (*n* = 3). The genotypes are displayed in two panels with identical axis scales to minimize line overlap and enhance figure readability.

**FIGURE 3:**
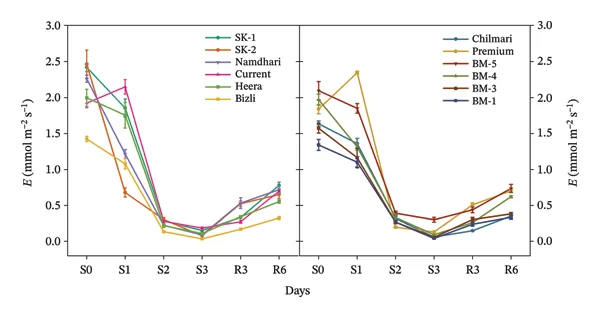
Changes in transpiration rate (*E*; mmol m^−2^ s^−1^) in the seedlings of 12 chilli genotypes on different days of waterlogging and recovery period. S0 denotes the day before WL stress; S1, S2, and S3 are the three consecutive days of WL stress; R3 and R6 denote third and sixth days of recovery period. Vertical bars are SD (*n* = 3). The genotypes are displayed in two panels with identical axis scales to minimize line overlap and enhance figure readability.

#### 3.2.2. Transpiration Rate (*E*)

The transpiration rate (*E*) in all chilli genotypes followed a similar pattern like *g*_*s*_ accompanying a drastic decline at day three (S3) of WL stress (Figure 3). Disparity in declination among the genotypes was visible; the reduction in *E* was highest in Chilmari and Heera (97%) compared to control. Like *g*_*s*_, the partial recovery of *E* was observed with varying degrees across the genotypes. The sensitive genotypes showed greater tendency to increase in *E* after a 6‐day recovery period as evidenced in BM‐4 (76%), BM‐3 (44%), Chilmari (30%), and Heera (23%) (Figure 3).

#### 3.2.3. Quantum Yield of PSII (*Φ*_*P* *S* *I* *I*_)

The quantum yield of PSII (*Φ*_*P* *S* *I* *I*_) varied significantly among the genotypes both under WL stress and recovery conditions (Supporting Table 1, Figure 4). The *Φ*_*P* *S* *I* *I*_ declined slowly till day 2 (S2) of WL stress while a drastic drop in *Φ*_*P* *S* *I* *I*_ was apparent on day 3 (S3) of WL (Figure 4). At 3^rd^ day of WL, the maximum reduction in *Φ*_*P* *S* *I* *I*_ was observed in Heera (38%), Bizli (38%), and Current (34%) compared to control, while a substantial recovery was observed in Bizli (31%), Heera (28%), and Namdhari (27%) in comparison to WL (Figure 4).

**FIGURE 4:**
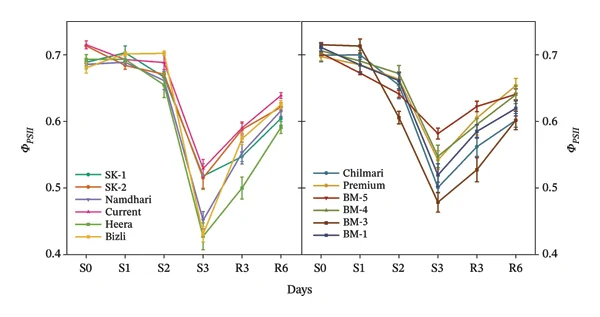
Quantum yield of PSII (*Φ*_*P* *S* *I* *I*_) in the seedlings of 12 chilli genotypes on different days of waterlogging and recovery period. S0 denotes the day before WL stress; S1, S2, and S3 are the three consecutive days of WL stress; R3 and R6 denote third and sixth days of recovery period. Vertical bars are SD (*n* = 3). The genotypes are displayed in two panels with identical axis scales to minimize line overlap and enhance figure readability.

#### 3.2.4. ETR

An irregular declining trend of ETR was noticed in all chilli genotypes during WL stress while a variable enhancement in ETR in recovery period was observed among the genotypes (Figure 5). The highest percent reduction in ETR was recorded in Chilmari (31%) followed by Bizli, BM‐1 (27%), and Namdhari (25%). The recovery of ETR was found higher in Chilmari, Heera, and Namdhari (14%) compared to WL after 6 days of recovery period (Figure 5). The results suggest that a recovery period of 6 days was not sufficient for full recovery in gaseous exchange and PSII efficiency in any of the tested genotypes.

**FIGURE 5:**
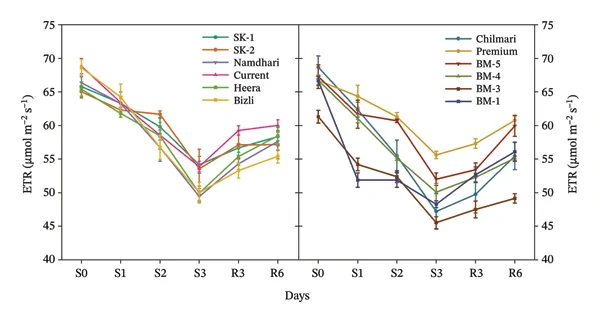
Electron transport rate (ETR; mol m^−2^ s^−1^) in the seedlings of 12 chilli genotypes on different days of waterlogging and recovery period. S0 denotes the day before WL stress; S1, S2, and S3 are the three consecutive days of WL stress; R3 and R6 denote third and sixth days of recovery period. Vertical bars are SD (*n* = 3). The genotypes are displayed in two panels with identical axis scales to minimize line overlap and enhance figure readability.

### 3.3. Biochemical Attributes

#### 3.3.1. Leaf Pigment Contents

The leaf pigment contents exhibited substantial variations among the treatments and genotypes (Supporting Table 1; Table 4). WL stress notably declined the leaf Chl a, Chl b, total Chl, and total Car contents in chilli plants compared to control (Table 4). WL‐mediated reduction in Chl a ranged from 13% (SK‐1 and Premium) to 36% (BM‐4) while a recovery from 7% (Current) to 31% (BM‐4) was apparent in contrast to control. During the recovery phase, the percent increase in Chl b was observed greatly in Heera (16%), Chilmari (11%), and SK‐2 (7%) compared to WL condition. Total Chl in most of the genotypes decreased during WL condition but restored during the recovery condition (Table 4). WL‐stressed leaves noticeably lost total Car contents from 13% to 33% compared to control which were enhanced in recovery period by 15%, 14%, 12%, and 10% in Premium, Chilmari, BM‐5, and SK‐1 compared to WL, respectively (Table 4). Lastly, the enhancement of pigment contents in SK‐1, SK‐2, Chilmari, Current, Heera, and Premium throughout the recovery period indicates their resilience to WL stress (Table 4).

**TABLE 4:** Chlorophyll a (Chl a), Chlorophyl b (Chl b), total chlorophyll (total Chl), and total carotenoids (total Car) contents in the leaves of 12 chilli genotypes grown in control, waterlogging (WL), and post‐stress recovery (Rec) conditions.

Genotypes	Treatments	Chl a (mg g^−1^ FW)	Chl b (mg g^−1^ FW)	Total Chl (mg g^−1^ FW)	Total Car (mg g^−1^ FW)
SK‐1	Control	1.42 ± 0.061 a‐e	1.10 ± 0.050 a‐e	2.53 ± 0.11 abc	2.78 ± 0.06 a‐d
	WL	1.24 ± 0.004 c‐i	1.06 ± 0.013 a‐g	2.30 ± 0.01 b‐j	2.33 ± 0.04 e‐l
	Rec	1.28 ± 0.015 a‐h	1.08 ± 0.025 a‐f	2.35 ± 0.01 a‐h	2.50 ± 0.05 c‐j

SK‐2	Control	1.40 ± 0.051 a‐f	1.06 ± 0.024 a‐g	2.45 ± 0.03 a‐e	2.84 ± 0.27 a‐d
	WL	1.06 ± 0.084 h‐l	0.98 ± 0.076 b‐g	2.10 ± 0.10 e‐l	2.02 ± 0.03 klm
	Rec	1.10 ± 0.059 g‐l	1.05 ± 0.035 a‐g	2.14 ± 0.09 d‐k	2.29 ± 0.10 f‐m

Namdhari	Control	1.26 ± 0.129 b‐h	1.10 ± 0.004 a‐e	2.31 ± 0.16 a‐i	3.10 ± 0.21 a
	WL	1.00 ± 0.086 jkl	0.99 ± 0.040 a‐g	2.10 ± 0.08 e‐l	2.54 ± 0.13 c‐j
	Rec	1.07 ± 0.050 h‐l	0.88 ± 0.028 e‐i	1.95 ± 0.02 jkl	2.58 ± 0.12 b‐h

Current	Control	1.48 ± 0.087 ab	1.18 ± 0.039 ab	2.66 ± 0.13 a	2.97 ± 0.14 ab
	WL	1.27 ± 0.047 a‐h	1.17 ± 0.008 abc	2.44 ± 0.06 a‐f	2.56 ± 0.08 b‐i
	Rec	1.37 ± 0.052 a‐f	1.16 ± 0.044 abc	2.53 ± 0.10 abc	2.71 ± 0.13 a‐f

Heera	Control	1.39 ± 0.104 a‐f	1.11 ± 0.058 a‐d	2.50 ± 0.05 a‐d	2.58 ± 0.16 b‐h
	WL	1.11 ± 0.032 g‐l	0.89 ± 0.067 d‐i	2.00 ± 0.10 h‐l	2.14 ± 0.17 i‐m
	Rec	1.25 ± 0.050 c‐h	1.07 ± 0.035 a‐g	2.32 ± 0.09 a‐i	1.98 ± 0.17 lm

Bizli	Control	1.32 ± 0.101 a‐g	1.06 ± 0.088 a‐g	2.38 ± 0.01 a‐g	2.90 ± 0.10 abc
	WL	1.05 ± 0.033 h‐l	0.98 ± 0.019 a‐g	2.06 ± 0.02 g‐l	2.21 ± 0.09 h‐m
	Rec	1.14 ± 0.022 g‐l	0.98 ± 0.032 a‐g	2.12 ± 0.01 e‐l	2.31 ± 0.14 f‐m

Chilmari	Control	1.45 ± 0.040 a‐d	1.15 ± 0.064 abc	2.60 ± 0.11 ab	2.66 ± 0.23 b‐g
	WL	1.10 ± 0.079 g‐l	0.95 ± 0.142 c‐g	2.05 ± 0.22 g‐l	2.13 ± 0.029 j‐m
	Rec	1.22 ± 0.148 e‐j	1.00 ± 0.197 a‐g	2.22 ± 0.35 c‐k	2.43 ± 0.18 d‐k

Premium	Control	1.49 ± 0.047 a	1.10 ± 0.016 a‐e	2.58 ± 0.07 ab	2.79 ± 0.13 a‐d
	WL	1.30 ± 0.035 a‐g	1.07 ± 0.005 a‐g	2.38 ± 0.04 a‐g	2.32 ± 0.10 e‐l
	Rec	1.23 ± 0.068 d‐i	0.85 ± 0.101 ghi	2.08 ± 0.03 g‐l	2.74 ± 0.13 a‐e

BM‐5	Control	1.40 ± 0.046 a‐f	1.10 ± 0.008 a‐e	2.49 ± 0.05 a‐d	2.77 ± 0.08 a‐d
	WL	1.17 ± 0.065 f‐k	0.97 ± 0.015 b‐g	2.16 ± 0.08 d‐k	2.26 ± 0.21 g‐m
	Rec	1.22 ± 0.057 d‐j	1.03 ± 0.054 a‐g	2.25 ± 0.11 b‐k	2.59 ± 0.10 b‐h

BM‐4	Control	1.46 ± 0.083 abc	1.20 ± 0.066 a	2.67 ± 0.02 a	2.87 ± 0.08 abc
	WL	0.94 ± 0.021 l	1.09 ± 0.178 a‐e	2.03 ± 0.20 g‐l	1.92 ± 0.01 lm
	Rec	1.01 ± 0.139 h‐l	0.97 ± 0.092 b‐g	1.98 ± 0.23 i‐l	2.12 ± 0.08 j‐m

BM‐3	Control	1.26 ± 0.043 b‐h	1.10 ± 0.002 a‐e	2.36 ± 0.04 a‐g	2.89 ± 0.06 abc
	WL	0.98 ± 0.020 kl	1.01 ± 0.057 a‐g	2.07 ± 0.00 g‐l	2.27 ± 0.07 g‐m
	Rec	1.11 ± 0.095 g‐l	0.68 ± 0.097 i	1.78 ± 0.00 l	2.51 ± 0.22 c‐j

BM‐1	Control	1.23 ± 0.026 d‐i	0.86 ± 0.041 f‐i	2.09 ± 0.07 f‐l	2.28 ± 0.11 f‐m
	WL	0.98 ± 0.064 kl	0.92 ± 0.021 d‐h	1.90 ± 0.08 kl	1.89 ± 0.17 m
	Rec	1.06 ± 0.045 h‐l	0.71 ± 0.015 hi	1.77 ± 0.06 l	2.03 ± 0.05 klm

*Note:* Values are mean ± SD. In a column, treatment means with dissimilar letters are significant at 5% levels of probability.

#### 3.3.2. MDA and H_2_O_2_ Contents in Leaf

The seedlings of all chilli genotypes grown in WL conditions encountered oxidative stress and significantly raised leaf MDA and H_2_O_2_ contents (Supporting Table 1; Figure 6). The leaf MDA contents ranged from 18.46 to 37.83 nmol g^−1^ FW depending on genotypes and treatments (Figure 6A). Compared to control, the MDA contents in waterlogged plants elevated by 29%–60%. The lowest MDA was found in SK‐1, Current, Premium, and BM‐3 in WL condition. During recovery period, MDA contents reduced in all genotypes compared to WL condition with a higher reduction in Namdhari (27%), Chilmari (25%), and BM‐1 (22%) genotypes (Figure 6A). Like MDA levels, the leaf H_2_O_2_ contents boosted by 24% (Current) to 46% (Chilmari) in WL condition (Figure 6B). After 6 days of recovery, the genotypes showed a variable falling trend in H_2_O_2_ levels accounted for 6% in Premium to 37% in Namdhari (Figure 6B). The greatest generation of H_2_O_2_ was found in Chilmari, Bizli, Namdhari, and BM‐1 while the genotypes Premium, SK‐1, and Current showed tolerance to WL by lowering the accumulation of leaf H_2_O_2_ contents (Figure 6B).

**FIGURE 6:**
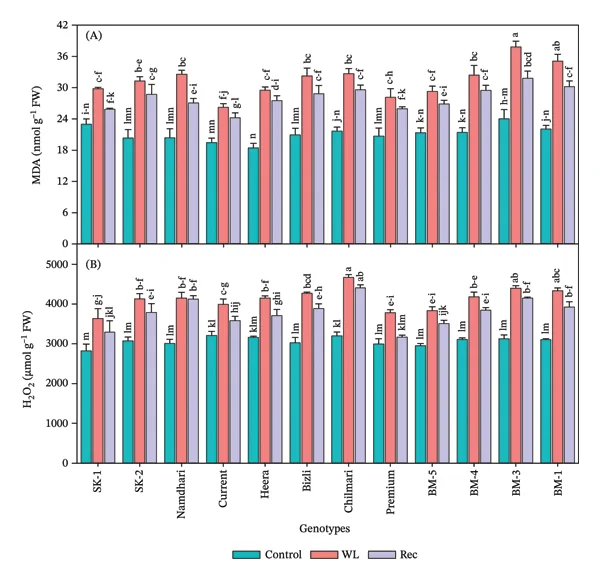
Leaf malondialdehyde (MDA; (A)) and hydrogen peroxide (H_2_O_2_; (B)) contents in 12 chilli genotypes grown in control, waterlogging, (WL) and post‐stress recovery (Rec) conditions. Vertical bars denote SD (*n* = 3). Treatment means with dissimilar letters within the genotypes denote significant differences at the 5% level of probability.

#### 3.3.3. Proline Content and TAC

The leaf proline contents and TAC markedly enhanced due to WL stress in all chilli genotypes (Supporting Table 1, Figure 7). Among tested genotypes, the highest proline content was recorded in waterlogged SK‐1 (82%), Current (80%), BM‐5 (74%), and Premium (72%) compared to control (Figure 7A). The leaf proline contents were further reduced in SK‐1, Current, and Premium by 54%, 65%, and 53% in recovery period compared to WL, respectively (Figure 7A). The percent reduction due to recovery across the genotypes varied from 5% to 25% compared to WL. During WL‐stress conditions, a notable fluctuation in TAC content was apparent while higher TAC was recorded in SK‐1, BM‐5, Current, SK‐2, and Premium which accounted for 83%, 87%, 82%, 78%, and 78% higher than the control, respectively (Figure 7B). Under post‐stress recovery, the TAC content of the leaves reduced by 63%, 65%, 54%, and 54% in SK‐1, Current, Premium, and BM‐5, respectively, compared to WL condition (Figure 7B).

**FIGURE 7:**
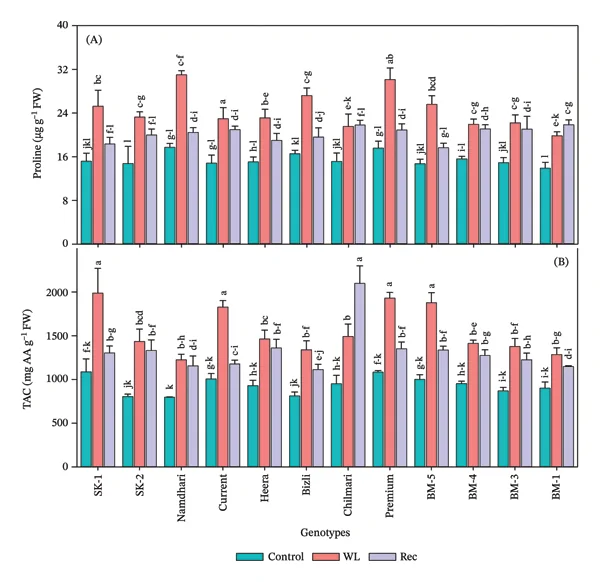
Leaf proline contents (A) and total antioxidant capacity (TAC (B)) in 12 chilli genotypes grown in control, waterlogging (WL), and post‐stress recovery (Rec) conditions. Vertical bars denote SD (*n* = 3). Treatment means with dissimilar letters within the genotypes denote significant differences at the 5% level of probability.

### 3.4. Hierarchical Clustering of Treatments and Traits

The immensity of WL tolerance among 12 genotypes was illustrated by two‐way hierarchical clustering heatmap considering the STI values of the 20 studied traits (Figure 8). The genotypes were classified into three clusters according to their response, as shown in rows. The tolerance and susceptibility of chilli genotypes were indicated by the higher STI (darker green) and lower STI (darker red) values, respectively. The genotypes were categorized into three clusters considering STI scores under WL stress where Cluster‐I (C‐I), C‐II, and C‐III consisting of seven, one, and four genotypes, respectively (Figure 8A). The 20 traits were further clustered into three groups following 2, 7, and 11 in Group‐1, Group‐2, and Group‐3, respectively. The four genotypes in C‐III (Current, Premium, SK‐1, and BM‐5) demonstrated greater WL tolerance by exhibiting higher STI values of the trait Group‐2 and Group‐3, whereas the genotypes in C‐I demonstrated sensitivity to WL stress (Figure 8A). Both genotypes and traits were further classified into three distinct clusters based on STI scores during the recovery phase (Figure 8B). The genotypes showed variability in response to post‐stress recovery depending on the traits studied (Figure 8B). Group‐1 traits decreased under recovery conditions reflecting lower STI scores and the Group‐2 and Group‐3 traits increased by exhibiting higher STI scores within C‐I, indicating enhanced WL tolerance (Figure 8B). C‐I consisted of five genotypes like SK‐1, Current, Premium, Chilmari, and BM‐5 contemplating tolerance to WL. In contrast, C‐II and C‐III exhibited moderate and severe susceptibility, respectively (Figure 8B). Hence, considering the STI scores in both WL and recovery conditions, the WL tolerance among the genotypes can be ranked as SK‐1 > Premium > Current > BM‐5 > Chilmari > BM‐3 > BM‐4 > BM‐1 > Namdhari > Bizli > SK‐2 > Heera.

**FIGURE 8:**
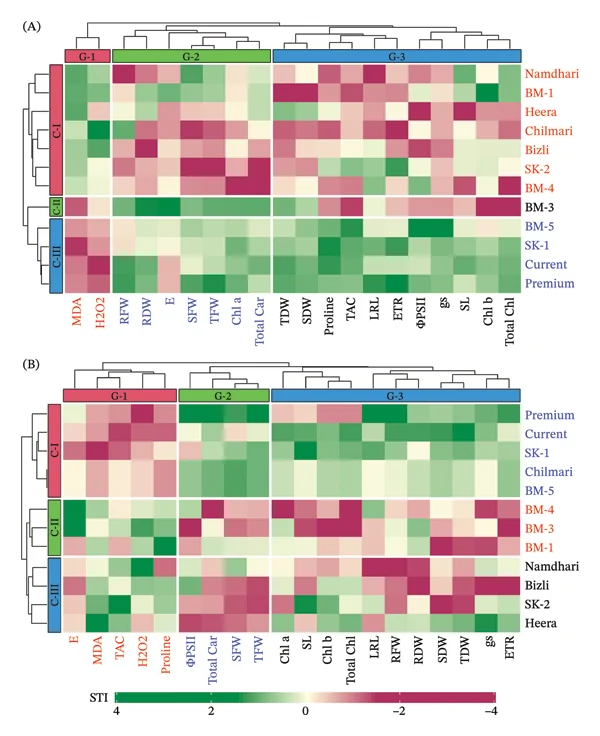
Heatmap using stress tolerance index (STI) values of studied traits in 12 chilli genotypes accompanying two‐way hierarchical clustering of genotypes and traits in waterlogging (A) and recovery (B) conditions. The columns and rows appear for traits and genotypes, respectively. The color gradient represents the corresponding STI values from a range of −4 (darker maroon indicating higher susceptibility) to 4 (darker green highlighting greater tolerance). Traits description: MDA, malondialdehyde; H_2_O_2_, hydrogen peroxide; SFW, shoot fresh weight; TFW, total fresh weight; Chl a, chlorophyll a; total Car, total carotenoids; RFW, root fresh weight; RDW, root dry weight; E, transpiration rate; *Φ*_*P* *S* *I* *I*_, quantum yield of PSII; *g*_*s*_, stomatal conductance; SL, shoot length; Chl b, chlorophyll b; total Chl, total chlorophyll; TDW, total dry weight; SDW, shoot dry weight; TAC, total antioxidant capacity; LRL, longest root length; ETR, electron transport rate.

### 3.5. PCA

To identify the variability of quantitative traits from a large complex dataset and the potential associations among the traits, a PCA‐biplot was made from the experimental dataset using 12 chilli genotypes and 20 diverse measured traits (Figures 9 and 10). The PCA biplot revealed that the first four principal components (PCs) scored eigenvalues of > 1 and cumulatively accounted for 84.3% of the total variation (Figures 9 and 10A,B). Among PCs, the first and second PCs explained maximum percentage, i.e., 70% of the total variation and, therefore, used to construct the PCA biplot with these PCs (Figure 9). PC1 contributed 50.1% of the entire variability and was mostly associated with MDA, ETR, H_2_O_2_, *g*_*s*_, pigment contents, RDW, and PSII efficiency attributes (Figures 9 and 10C). The PC2, on the other hand, accounted for 19.1% of the total variability among traits and mainly linked with SDW, TDW, TFW, RFW, and proline content (Figures 9 and 10D). The PCA biplot displayed a distinct separation among the control, WL, and Rec treatments based on the measured traits where control and WL observations remained a long distance apart while Rec retained between control and WL (Figure 9). In the PCA biplot, the physiological parameters (antioxidative defense) were in opposite directions with biochemical traits and the morphological attributes lied between them explaining that the growth of chilli seedlings was regulated through integrated physiochemical processes (Figure 9).

**FIGURE 9:**
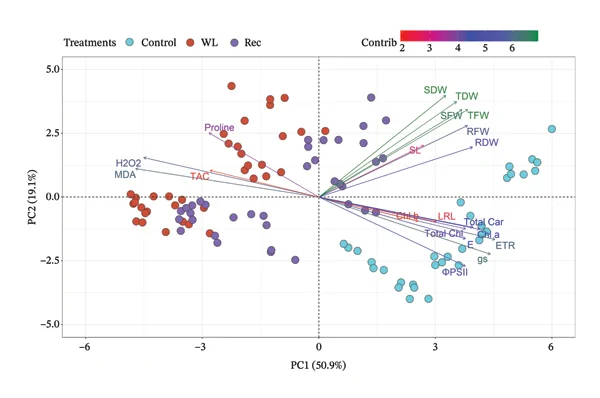
Principal component analysis (PCA) biplot explaining the variability among three growth conditions (control, waterlogging (WL), and post‐stress recovery (Rec)) contemplating 20 measured traits in 12 chilli genotypes. The arrow length and color intensity (red to blue) highlight the extent of absolute contribution of the traits to PC1 and PC2. The PC1 and PC2 contributed 50.9% and 19.1% of total variation, respectively. Trait descriptions are same as Figure 10.

**FIGURE 10:**
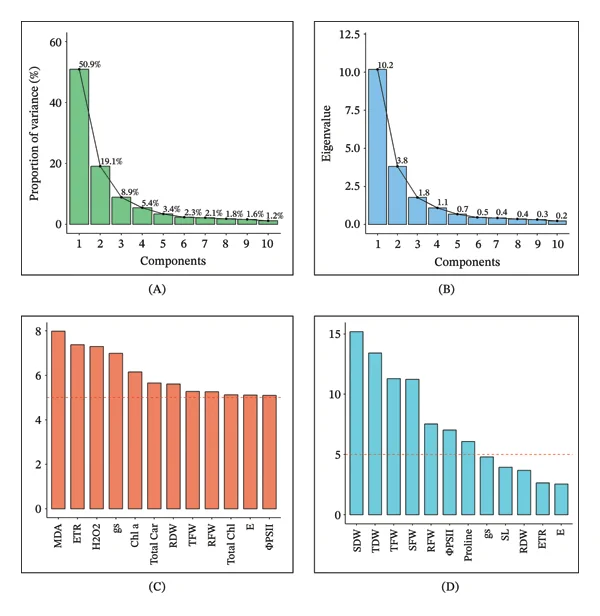
Top 12 principal components (PCs) extracted from PCA showing percent contributions (A), eigenvalues (B), contributing characters to PC1 (C), and contributing characters to PC2 (D). PC threshold limit of contribution is highlighted by red‐dashed lines across the bars.

## 4. Discussion

WL is among the most lethal abiotic stresses limiting chilli (*Capsicum annuum* L.) growth and yield worldwide including Bangladesh where erratic rainfall, flash flood, and poor drainage frequently cause WL [36]. Chilli plant is highly sensitive to WL stress and shows diverse morphological, physiological, and biochemical alterations when subjected to WL stress [17, 34]. The present study investigated 12 chilli genotypes to evaluate their morpho‐physiological and biochemical responses under short‐term WL stress and post‐stress recovery.

In this study, the WL‐induced reduction in root‐shoot length in all chilli genotypes may be due to an impairment of cell division and elongation because of restricted oxygen diffusion to root tissues resulting in a decrease of root‐shoot fresh weight and dry mass [48]. However, the reduction in root biomass was more detrimental than in shoot biomass, reflecting the instant impact of hypoxia on root metabolism. These findings are consistent with other earlier reports that oxygen deprivation restricts cell division and elongation in root and shoot meristems [49]. It impaired ATP synthesis and hormonal imbalance caused by hypoxia [50]. In this study, the genotypes Current, Premium, BM‐5, and SK‐1 exhibited relatively lower reduction in RL and SL, suggesting better root aeration capacity and tissue oxygenation. Previous studies revealed that tolerant rice and maize cultivars maintained higher root elongation under deficit oxygen conditions through the formation of aerenchyma and adventitious roots [51]. The cultivars studied in the present study differentially responded in growth attributes during the recovery phase showing variability in WL tolerance across them. The genotypes Current, Premium, BM‐5, and SK‐1 displayed improved biomass accumulation during the recovery phase. Xu et al. [52] reported that tolerant wheat cultivars demonstrated better regrowth after WL due to rapid antioxidative defense and membrane stabilization mechanisms.

The WL‐induced depletion of soil oxygen may disrupt water uptake resulting in stomatal closure as a defense mechanism against water loss leading to a marked decline in transpiration rate [14, 53]. The stomatal closure due to WL increased CO_2_ diffusion resistance impeding the rate of photosynthesis and thereby reduced plant growth [13]. In this study, *g*_*s*_ and *E* decreased sharply (closer to 0) in comparatively less tolerance genotypes (Bizli, Namdhari, and Heera) under short‐term WL, indicating impaired stomatal function with limited photosynthetic efficiency. Conversely, the genotypes Current, Premium, SK‐1, and BM‐5 maintained comparatively higher *g*_*s*_ pattern than others, suggesting lower stomatal‐induced photosynthetic inhibition and, therefore, maintained better growth under hypoxic conditions. A greater variation in *g*_*s*_ and *E* was observed among different *Capsicum* species where *C. pubescens* and a wild *C. baccatum* showed increased *g*_*s*_ and *E* indicating greater tolerance to WL [17].

The estimation of Chl a fluorescence is a fast, reliable, and nondestructive technique that allows to evaluate the extent of plants’ tolerance to abiotic stresses [54]. The commonly used Chl *a* fluorescence parameters for phenotyping or screening of plants are maximum photochemical efficiency of PSII (*F*_*v*_/*F*_*m*_), quantum yield of PSII (*Φ*_*P* *S* *I* *I*_), ETR, nonphotochemical quenching (NPQ), and photochemical quenching (*qP*) [55, 56]. The stomatal closure due to WL reduces the rate of photosynthesis by limiting CO_2_ diffusion, which lowers ATP and NADPH demand and subsequently decreases linear electron transport leading to reduced *Φ*_*P* *S* *I* *I*_ and ETR [57]. The thylakoid reactions might also be affected by WL stress due to overproduction of ROS at the PSI and PSII sites disrupting the ETC resulting in *Φ*_*P* *S* *I* *I*_, NPQ, *qP,* and ETR reduction [58]. In alignment with this study, the *Φ*_*P* *S* *I* *I*_ and ETR markedly declined with the increase of WL duration in peanut and cape gooseberry plants affecting the rate of photosynthesis [59, 60].

Prolonged WL diminishes chlorophyll synthesis and hampers light absorption leading to a marked decline in the rate of photosynthesis [15]. As a result, leaf yellowing and peeling occur and accelerate leaf senescence [61]. The contents of photosynthetic pigments in leaves such as chlorophyll and carotenoids are an important indicator of susceptibility or tolerance of plants to abiotic stress including WL [59]. In the current study, a significant reduction in Chl a, Chl b, total Chl, and total Car as well as PSII efficiency (*Φ*_*P* *S* *I* *I*_ and ETR) appeared in all chilli genotypes due to WL stress. However, the leaf pigment reduction and PSII efficiency were greater in sensitive seedlings as compared to tolerant chilli seedlings. The degradation of leaf chlorophyll under WL stress could be attributed to decrease in oxygen availability, increased activity of chlorophyllase enzyme, disruption of chloroplast ultrastructure, and overproduction of ROS [22, 62, 63]. Rho et al. [34] observed a considerable decline in photosynthetic pigments and composition of the light harvesting complex after 3 days of WL which further led to a decline in SPAD (index of chlorophyll content) values at 5 days of WL in several *Capsicum* species. The leaf chlorophyll and carotenoid contents gradually declined in a WL‐tolerant *C. frutescence* species with the increase of WL duration while a subsequent recovery of leaf pigment contents was observed [36]. Higher chlorophyll and carotenoid contents in tolerant chilli genotypes suggest lesser inhibition of chlorophyll degradation maintaining greater photosynthetic capacity in tolerant genotypes than the susceptible ones [64]. Like this study, selection of genotypes to WL tolerance based on leaf photosynthetic pigment contents at different WL periods and recovery has been performed in many other crops like sesame [65], black gram [66], soybean [67], and tomato [68].

Insufficient oxygen under WL conditions may lead to increase in intracellular ROS such as superoxide radicals (•O_2_), hydroxyl radicals (•OH), and hydrogen peroxide (H_2_O_2_) [18, 69]. These ROS have strong oxidative properties causing lipid peroxidation, damage to proteins, DNA, cell membranes, and organelles and ultimately cause cell death [19, 70]. MDA is a reliable biochemical marker for lipid peroxidation in plants indicating the extent of oxidative stress. A substantial increase in MDA and H_2_O_2_ levels was recorded in all studied chilli genotypes under WL stress reflecting overproduction of ROS indicating membrane damage. The genotypes Premium, Bizli, and SK‐1 exhibited greater tolerance showing relatively lower MDA and H_2_O_2_ levels while BM‐4, Namdhari, and Heera showed sensitivity to WL, scoring higher levels of MDA and H_2_O_2_. Similar findings in relation to the increment of MDA and H_2_O_2_ levels under WL stress have been reported in previous studies in chilli and other crop plants [12, 34, 64, 71, 72]. Anee et al. [65] claimed an increased level of MDA and H_2_O_2_ after 8 days of WL in *Sesamum indicum* while injury to lipid membranes in chloroplast are reported to appear in susceptible barley [73] and maize hybrids [74].

To cope with ROS‐mediated oxidative injury under WL stress, plants activate both their enzymatic and nonenzymatic defense mechanisms [29]. Proline accumulation is a well‐known defense response to abiotic stress, serving as an effective osmoprotectant and potential ROS scavenger [75]. In the present study, proline content remarkably increased across all genotypes where Premium, Bizli, and BM‐1 had the highest accumulation. Proline plays key role in maintaining cellular osmotic balance, stabilizing proteins and membranes, and protecting enzymes from denaturation [75]. Less tolerant chilli genotypes (BM‐4, Heera, and Namdhari) accumulated lower proline corresponding with enhanced MDA and H_2_O_2_ levels experiencing greater oxidative stress. This inverse relationship between proline and ROS in this study indicates that tolerant genotypes were prevented from oxidative injury through enhanced accumulation of proline. Proline contents increased in several *Capsicum* species in varying magnitude under WL stress with a wild pepper species increasing proline content by 19.7% compared to control [17].

The TAC reflects the overall potential of the plant’s antioxidant system to counteract reactive excess generation of ROS under stress. The higher TAC values in Premium, Current, SK‐1, and Chilmari during both stress and recovery stages suggest more activated defense system inhibiting lipid peroxidation and cellular injury. In contrast, Heera, Bizli, and Namdhari genotypes displayed comparatively lower TAC, making them more susceptible to ROS accumulation and membrane damage. The enzymatic antioxidants such as SOD, CAT, POD, GPX, and ADH activities increased in pepper plants after 5 days of WL stress [32]. Tolerant maize genotypes subjected to WL displayed elevated SOD, POD, and CAT activities to withstand oxidative injury [76]. Similarly, enhanced SOD and CAT activities were reported in the WL‐resistant JN01 and JZ31 sorghum lines [77]. Luan et al. [73] revealed that both tolerant and sensitive barley genotypes exhibited higher activities of SOD, POD, and CAT when exposed to WL stress for 21 days. Enhanced activity of antioxidative enzymes during post WL stress increases aquaporin activity, root hydraulic conductivity, transpiration rate, and photosynthesis in *Brassica napus* [78].

The overall performance of the studied chilli genotypes based on 20 traits under WL stress clearly indicates that tolerance is a complex, multifactorial trait involving the coordinated function of morphological, physiological, and biochemical mechanisms. Oxygen deprivation under WL initially hindered root activity restricting uptake of water and nutrients and consequently suppressing root and shoot growth [42]. The abridged root hydraulic conductivity prompted stomatal closure leading to limit transpiration, restrict CO_2_ diffusion, and decline photosynthetic efficiency as evidenced by lower stomatal conductance, PSII efficiency, and pigment contents in this study. Simultaneously, hypoxia triggered excess generation of ROS in the mitochondria and chloroplast resulting in enhanced MDA and H_2_O_2_ levels that further damaged photosynthetic apparatus and cellular membranes. Tolerant genotypes were able to cope with oxidative injury through higher proline accumulation and greater antioxidant capacity thereby protecting membrane integrity, stabilizing photosynthetic performance, and maintaining better growth with increased biomass under WL.

The post‐WL recovery is a critical determinant of plant survival and productivity because the normal physiological and metabolic functions may not immediately reestablish by the restoration of aerobic conditions. A considerable variation in recovery ability among studied chilli genotypes were noticed in this study suggesting that recovery potential is a crucial component of WL tolerance. Immediately after drainage, the integrated morphological, physiological, and biochemical responses enabled better performing genotypes to a rapid restoration of root functioning, gas exchange, pigment synthesis, and biomass accumulation. Less tolerant genotypes, in contrast, displayed persistent oxidative damage with restricted growth and limited physiological recovery. Although a 6‐day recovery in this study offered valuable insights into the early post‐WL responses of chilli seedlings, it was inadequate to attain complete recovery of growth, photosynthetic performance, and redox homeostasis in almost all genotypes. These traits remained markedly away from the control state which indicates that the negative impacts of transient hypoxia persisted beyond the 6 days of recovery period. The restoration of root activity, reactivation of photosynthesis, and repairment of oxidative damage are the overall mechanisms of recovery from WL which may require a longer period depending on stress severity and genotype [36, 79]. In a previous study, SRC‐517 and BARI Morich‐2 genotypes exhibited better recovery showing faster root and shoot growth after aeration restoration of seven days [5]. This recovery ability may be linked with rapid restored carbohydrate mobilization and reactivation of root metabolism during re‐oxygenation [80]. Saptiningsih et al. [36] reported that the recovery potential of *Capsicum frutescence* depends on WL duration, while complete recovery in wilting, root damage, stomatal closure, photosynthetic pigment degradation, and reduced plant biomass following WL extends beyond 1 week to 4 weeks. Therefore, while the present study effectively captured the initial recovery responses and differentiated genotypic resilience, longer recovery periods should be considered in future studies to better characterize the long‐term recovery capacity and adaptive mechanisms of chilli genotypes under WL stress.

The findings of the present study revealed substantial variations in WL tolerance and post‐WL recovery among local, hybrid, and inbred chilli genotypes reflecting that tolerance to WL is markedly genotype‐dependent. Hybrid cultivar Premium exhibited better growth and photosynthetic performance with reduced oxidative damage followed by quick recovery which may be attributed to greater genetic heterogeneity and hybrid vigor than other local and inbred cultivars as well as other studied hybrids. Conversely, local landraces including Current and SK‐1 displayed greater tolerance to WL and subsequent recovery considering most studied traits suggesting that they possess wider genetic makeup and key adaptive traits that have evolved under local environmental conditions. Inbred cultivars showed susceptibility to WL displaying impaired growth and photosynthesis with enhanced oxidative damage. Similar genotypic variation in flooding tolerance has been reported among *Capsicum* species due to differences in their physiological, anatomical, and genetic characteristics [5, 7, 36, 81]. For instance, *Capsicum frutescens* exposes greater WL tolerance than *C. chinense*, as reflected by its higher stomatal behavior and photosynthetic performance under waterlogged conditions [82]. Thus, the findings of this study suggest that both hybrid and locally adapted germplasm constitute valuable genetic resources for developing WL‐tolerant chilli cultivars and highlight the importance of exploiting genetic diversity in future breeding programs.

Various indices have been developed to quantify plant performance under abiotic stress conditions including STI, tolerance index (TOL), mean productivity (MP), stress susceptibility index (SSI), etc. [83, 84]. Among these, STI has been widely used because of its effective and simultaneous selection of genotypes that perform well both under stress and nonstress conditions [46, 85–87]. STI identifies genotypes that maintain high productivity in both environments, and it combines tolerance and productivity in a single value, as well as relative tolerance and yield potential. In this study, normalized STI values were integrated to construct hierarchical clustering of genotypes to provide a robust framework and biologically meaningful evaluation of genotypes in response to WL stress. While STI quantitatively ranks genotypes based on their performance under both control and stress conditions, hierarchical clustering classifies genotypes according to their overall similarity across multiple morphological, physiological, and biochemical traits. This complementary approach not only validates the ranking obtained from STI but also reveals the underlying patterns of stress adaptation and recovery. Genotypes with high STI values that consistently cluster together are more likely to possess stable and coordinated tolerance mechanisms, including maintenance of growth, photosynthetic capacity, and antioxidant defense under WL. Moreover, hierarchical clustering facilitates the identification of genetically diverse tolerant groups, providing valuable information for parent selection in breeding programs. In the present study, under both WL and recovery period, Premium, Current, and SK‐1 genotypes within Cluster‐I and Cluster‐III exhibited superior tolerance, respectively, as indicated by steadily higher STI values across all studied traits. In contrast, BM‐1, BM‐3, BM‐4, and Namdhari genotypes displayed intense susceptibility to WL stress.

In the PCA biplot, strong positive loadings were exhibited among the morphological traits (root‐shoot length, root‐shoot biomass) as well as physiological attributes (*g*_*s*_, *E*, *Φ*_*P* *S* *I* *I*_, ETR, and pigments) reflecting that growth and photosynthetic performance are the key determinants of WL tolerance. These traits were positively linked with PC1 while the oxidative stress markers including H_2_O_2_ and MDA contents were negatively associated with PC1 indicating that enhanced oxidative injury is a distinctive feature of susceptible genotypes. Tolerant genotypes were positively correlated with proline content and TAC suggesting that stimulated antioxidative defense system plays a pivotal role in limiting ROS‐induced cellular damage and facilitating post‐stress recovery. The distinct separation of control and WL samples on the PCA biplot demonstrates that WL caused substantial alterations in morpho‐physiological and biochemical traits. Recovery samples were situated between control and WL revealing partial recovery of the seedlings implying that 6 days of recovery was not enough for complete return to the control state. The clear discrimination between tolerant and susceptible groups summarizing that better‐performing genotypes under WL conditions were closely related to improved growth, greater photosynthetic performance, and stronger antioxidative defense. whereas susceptible genotypes were associated with elevated oxidative damage as well as declined growth and photosynthesis. The strong positive linkage among traits further suggests that these attributes are interrelated and collectively contribute to WL tolerance rather than acting independently.

In breeding perspectives, from a large number of correlated traits, PCA effectively identifies the key selection criteria, thereby minimizing redundancy and improving screening efficiency. Traits exhibiting high loadings on the PCs can serve as reliable phenotypic markers for indirect selection of WL‐tolerant genotypes. The traits with the longest vector lengths on the PCA biplot can be considered as most informative selection criteria because they contributed most to the observed variation among treatments. Among 20 traits studied, the morphological traits such as SDW and TDW; the physiological traits including *g*_*s*_, *E*, ETR, and Chl a; and the biochemical traits such as H_2_O_2_ and MDA contents contributed more to depicting the variation across the genotypes and therefore these traits could be treated as key selection criteria. Moreover, the clear partition of tolerant and susceptible genotypes on the PCA biplot facilitates the identification of elite donor parents possessing complementary adaptive traits for hybridization. Therefore, integrating PCA with STI and cluster analysis enhances the accuracy of genotype assessment and provides a robust framework for developing WL‐resilient chilli cultivars through marker‐assisted or conventional breeding programs.

## 5. Conclusions

The tolerance potential of 12 chilli genotypes to short‐term WL and recovery was assessed based on their growth, physiological performance, and tolerance to oxidative damage. Among them, the genotypes Sariakandi‐1, Premium, BARI Morich‐5, and Current exhibited superior tolerance displaying lower reductions in growth, pigment levels, stomatal regulation, and PS functions with enhanced redox stability. None of the genotypes recovered completely after the 6‐day recovery period; however, the potentially tolerant genotypes exhibited relatively faster and greater recovery. The study identified key traits such as stomatal conductance, quantum yield of PSII, plant biomass, and oxidative stress markers (MDA and H_2_O_2_ contents) that can be considered as selection criteria offering a foundation for developing WL‐tolerant chilli cultivars through breeding programs. However, the findings of this study require further validation under field conditions before they can be incorporated into practical breeding and cultivation strategies. Moreover, long‐term WL responses and recovery potential in chilli across vegetative and reproductive stages emphasizing anatomical and molecular adaptations should be investigated.

## Author Contributions

Md. Sabibul Haque: conceptualization, funding acquisition, project administration, formal analysis, visualization, writing–review and editing, and supervision; Zarin Shaima: writing–original draft, methodology, investigation, and data curation; Md. Fazle Rabbi: methodology, investigation, visualization, and data curation; Hafsa Tasnim: methodology and investigation; Atia Iffat: methodology and investigation; Susmita Biswas: writing–original draft, methodology, and investigation; Md. Nesar Uddin: writing–review and editing and supervision. Md. Alamgir Hossain: writing–review and editing and supervision.

## Funding

The research was supported by a research grant from Bangladesh Agricultural University (Project No. 2024/09/BAU).

## Conflicts of Interest

The authors declare no conflicts of interest.

## Supporting Information

Additional supporting information can be found online in the Supporting Information section.

## Supporting information


**Supporting Information** Supporting Table 1: Analysis of variance (ANOVA) for several measured traits in 12 chilli genotypes grown in different growth conditions.

## Data Availability

Data are available upon request from the corresponding author.
