# Supplementary material for: Physiological and Biochemical Screening of Chilli (Capsicum annuum L.) Genotypes to Short‐Term Waterlogging Stress at the Seedling Stage
Source: Scientifica (Cairo). 2026 Sep 30;2026:4290084. doi: 10.1155/sci5/4290084 (PMC13627861; doi:10.1155/sci5/4290084)
Supplement: Supplementary file 1 [file SCI5-2026-4290084-s001.docx]

**Supporting Table 1:** Analysis of variance (ANOVA) for several measured traits in twelve chilli genotypes grown in different growth conditions.

| **Traits** | **Sources of variation** | | | LSD for interactions (5% LoS) |
| --- | --- | --- | --- | --- |
|  | Genotype (G) | Treatment (T) | Gx T |  |
| Longest Root Length (LRL) | *** | *** | ** | 2.02 |
| Shoot Length (SL) | *** | *** | NS | 2.22 |
| Root Fresh Weight (RFW) | *** | *** | *** | 32.20 |
| Shoot Fresh Weight (SFW) | *** | *** | *** | 154.53 |
| Root Dry Weight (RDW) | *** | *** | *** | 9.41 |
| Shoot Dry Weight (SDW) | *** | *** | ** | 25.83 |
| Total Fresh Weight (TFW) | *** | *** | *** | 161.10 |
| Total Dry Weight (TDW) | *** | *** | *** | 29.83 |
| Stomatal Conductance (*g_s_*) | *** | *** | *** | 0.03 |
| Transpiration Rate (*E*) | * | *** | * | 1.51 |
| Quantum Yield of PSII (*Φ_PSII_*) | *** | *** | *** | 0.07 |
| Electron Transport Rate (*ETR*) | *** | *** | NS | 9.23 |
| Chlorophyll a | *** | *** | * | 0.23 |
| Chlorophyll b | *** | *** | *** | 0.23 |
| Total Chlorophyll | *** | *** | *** | 0.36 |
| Total Carotenoid | *** | *** | *** | 0.43 |
| Malondialdehyde (MDA) | *** | *** | *** | 4.60 |
| Hydrogen peroxide (H_2_O_2_) | *** | *** | *** | 372.41 |
| Proline content | *** | *** | *** | 5.47 |
| Total Antioxidant Capacity (TAC) | *** | *** | *** | 305.87 |

***= Significant at 0.1% level, **= Significant at 1% level, *= Significant at 5% level, NS= Non-significant. LSD = Least Significant Difference, LoS = Level of Significant
